# Effects of Coach Behaviors on Training Engagement and Disaffection of Chinese Youth Male Basketball Players: The Role of Basic Psychological Needs and Psychological Capital

**DOI:** 10.5114/jhk/209808

**Published:** 2025-11-20

**Authors:** Yuanchang Li, Guoxing Li, Wenwei Huang, Qianyu Zeng, Junxing Liao, Jian Sun, Xiaohui Hou

**Affiliations:** 1 School of Athletic Training, Guangzhou Sport University, Guangzhou, China.; 2 Guangdong Provincial Key Laboratory of Human Sport Performance Science, Guangzhou Sport University, Guangzhou, China.

**Keywords:** self-determination theory, Chinese basketball, coach behavior, athlete, training, motivation, performance, structural equation model

## Abstract

This study investigated the relationship between coaches' behavior and athletes' engagement and disaffection in the Chinese sports training context, and examined the mediating role of basic mental needs and psychological capital. Athletes (N = 485) completed a questionnaire to assess the study variables. The results showed significant direct influence of coach supportive behavior on athletes' training engagement and coach controlling behavior on athletes' training disaffection. In addition, Bootstrap analyses highlighted the significant mediating role of basic mental needs and psychological capital in the relationship between coaches' behavior (supportive and controlling behavior) and athletes' training engagement and disaffection. Findings support theoretical hypotheses emphasizing that coaches should enhance supportive behaviors and reduce controlling behaviors in Chinese youth basketball training, which is not only of great significance to cultivate athletes' positive psychological capital, but also of great importance to promote athletes' engagement as well as avoid disaffected emotions and behaviors during training.

## Introduction

Participating in organized physical activity during adolescence has been found to be connected to higher physical activity and better subjective health in early adulthood (Logan et al., 2019). However, in high-level sports, athletes are generally exposed to high training loads, putting them under competitive and performance pressure. They also need to balance sports, studies, and interpersonal relationships (di Luzio et al., 2020), which might cause athletes to experience psychological and social obstacles, sometimes pushing them to quit (Bédard Thom et al., 2021). This is the main reason for continuous decline of the competitive level of China's basketball in recent years. Therefore, to attract more teenagers to participate in basketball, it is essential to enable participants to experience high enjoyment levels to stimulate their training motivation.

### Engagement and Disaffection in Sport

In sports and other achievement contexts, engagement and disaffection are considered indicators to measure motivation quality (Rodríguez-Medellín et al., 2020). Engagement combines behavioral and emotional dimensions. It contains two dimensions, with behavioral engagement reflecting efforts, attention and perseverance during the initiation and implementation of activities, and emotional engagement referring to the states associated with emotions in an activity, such as interest, enjoyment and enthusiasm (Zamarripa et al., 2021). The opposite of engagement is disaffection, which is defined as passive participation, showing a high degree of negative emotions and psychological withdrawal resulting in the formation of destructive social environments, hindering people from finding new motivation sources (Rodríguez- Medellín et al., 2020). It also includes behavioral and emotional dimensions.

Behavior disaffection includes core disengagement behavior, that is, passivity, non-effort, giving up and ritual participation, which refers to inattention and going through motions. On the other hand, emotional disaffection includes emotional disorders that reflect weakness (fatigue, sadness, boredom), alienation (depression, anger), and stressful participation (anxiety) (Curran and Standage, 2017).

### Relationship between Coach Behaviors and Engagement/Disaffection

Coaches play vital roles in cultivating positive or negative experience among athletes. Within the framework of self-determination theory (SDT), coach behavior could be divided into two categories of supportive and controlling behaviors (Murillo et al., 2022). Previous research has shown that coach supportive behaviors have positive effects on promoting the autonomy motivation and engagement of athletes (De Muynck et al., 2019), and reducing their disaffection (Curran et al., 2014, 2016). Conversely, when controlling, coaches actively adopt various strategies to limit and interfere with the behaviors, thoughts or feelings of athletes (Reynders et al., 2020), which increases their controlling motivation and decreases their engagement (Chu et al., 2021; Fenton et al., 2016).

Furthermore, existing research has revealed that controlling behavior is the main factor creating the disaffection, disengagement and even withdrawal of youth athletes (Reynders et al., 2020). This is because coaches always impose external conditions on athletes, which results in more controlled motivation, giving rise to disaffection, boredom, unhappiness, effort decline and other non-adaptive emotions and behaviors (Curran et al., 2016, Patterson et al., 2025). In addition, the opportunity to ask questions provided by PE teachers negatively predicts the disaffection of students (Patall et al., 2018), and lower levels of perceived teacher support results in student disengagement from the classroom (Van den Berghe et al., 2015). Accordingly, we proposed the following hypotheses:

H1a: Coach supportive behavior has a positive effect on training engagement.
H1b: Coach controlling behavior has a negative effect on training engagement.
H1c: Coach controlling behavior has a positive effect on training disaffection.
H1d: Coach supportive behavior has a negative effect on training disaffection.

### The Mediating Role of Basic Psychological Needs

SDT states that the social environment can improve the internal motivation of individuals and internalize their external motivation by satisfying the three basic psychological needs (i.e., BPNs; autonomy, relatedness, and competence needs), ensuring optimal functioning and healthy growth of individuals. Similarly, individuals might also think that the realization of these three psychological intermediaries is frustrating, leading to non-adaptive motivational outcomes (Murillo et al., 2022).

Previous studies have shown that coach supportive behavior can positively predict psychological needs satisfaction (BPNS) of athletes and negatively predict their psychological needs frustration (BPNF; Trigueros et al., 2019). However, SDT points out that when people are in overly controlling, challenging or repulsed environments, these BPN processes are obstructed, possibly resulting in lower BPNS and higher BPNF (Morales-Sánchez et al., 2020). BPNS, in turn, stimulates intrinsic motivation, showing higher participation and lower dissatisfaction (Curran et al., 2016). However, BPNF positively correlates with negative emotions, disaffection and controlling motivation, and has a negative explanation for exercise persistence (Sevil-Serrano et al., 2021). Thus, the following assumptions were made:

H2a: BPNS mediates the relationship between coach behavior and training engagement.
H2b: BPNF mediates the relationship between coach behavior and training engagement.
H2c: BPNS mediates the relationship between coach behavior and training disaffection.
H2d: BPNF mediates the relationship between coach behavior and training disaffection.

### The Mediating Role of Psychological Capital

Psychological capital (PsyCap) is regarded as the positive psychological states of individuals and includes four dimensions: self-efficacy, optimism, hope, and resilience, and is believed to have the potential to be managed and developed for the individual’s optimal flourishing (Burhanuddin et al., 2022). PsyCap among athletes indicates positive psychological states, which are related to their sense of control, adaptive coping, and agentic goal pursuit in development processes (Kim et al., 2020). In a competitive sport environment, the PsyCap of athletes is highly associated with the promotion of positive attitudes in sport teams (McDowell et al., 2018), athletes' higher engagement (Kim et al., 2020), and their fight against negative emotions and athletic burnout (Chang et al., 2019).

PsyCap, as a developmental state, is ‘state-like’ in nature and open to development, especially as leadership behavior is one of the primary antecedents of followers' PsyCap (McDowell et al., 2018). In sports, positive coach behaviors plays a critical role in promoting athletes’ self-efficacy (Di Corrado et al., 2023), optimism (deBeaudrap et al., 2017), and resilience to persist through challenges and adversity (Zhang et al., 2023), while negative coach behaviors were correlated with the goal setting, the confidence level and self-efficacy of athletes (de Albuquerque et al., 2021). Accordingly, the research proposed the following hypotheses:

H3a: PsyCap mediates the relationship between coach supportive behavior and training engagement.
H3b: PsyCap mediates the relationship between coach supportive behavior and training disaffection.
H3c: PsyCap mediates the relationship between coach controlling behavior and training engagement.
H3d: PsyCap mediates the relationship between coach controlling behavior and training disaffection.

### Chain Intermediary Effects of BPNs and PsyCap

In terms of the properties of PsyCap, it refers to an individual's positive psychological development. Therefore, we consider it to be operationalization of “ongoing psychological growth” in this paper. Within SDT, needs are defined as universal necessities specifying “innate psychological nutriments that are essential for ongoing psychological growth, integrity, and well-being”. From this perspective, both BPNs and PsyCap are inherent to human nature, and BPNs operate to promote positive psychological development and full realization of human potential across the lifespan (Ryan and Deci, 2017).

According to SDT, people feel more energy when they believe that the social environment satisfies their needs for competence, autonomy, and relatedness, and energy decreases when they perceive the social environment to thwart the fulfillment of those needs. However, at present, in sports, especially in the Chinese context, the construction and application of PsyCap are relatively novel, and the explicit link between the mechanism of BPNs and nurturing PsyCap is missing. Nevertheless, research in other fields has shown that promoting the ways and means to achieve autonomy, competence and relatedness can help individuals in developing PsyCap resources such as hope, efficacy, resilience and optimism (Plessis and Altintas, 2024). Building on this idea and the above discussion of coach behaviors, we suggest that different types of coach behaviors are antecedents to athletes experiencing increased or decreased energy, which in turn correlates with the athletes' positive mental development or PsyCap and further associates with their engagement and disaffection. Given the discussion thus far, we hypothesized that:

H4a: BPNs and PsyCap act as chain mediators between supportive behavior and training engagement
H4b: BPNs and PsyCap act as chain mediators between supportive behavior and training disaffection.
H4c: BPNs and PsyCap act as chain mediators between controlling behavior and training engagement.
H4d: BPNs and PsyCap act as chain mediators between controlling behavior and training disaffection.

In summary, the present study sought to contribute to a more detailed knowledge of the relationships between different coach behaviors and athletes' BPNs, PsyCap, training engagement and disaffection in the Chinese cultural context. To this end, this study also explored the mediating role of athletes' BPNs and PsyCap in the association between coach behaviors and athletes' training engagement and disaffection. To the best of our knowledge, no previous studies have examined a combination of such variables in a comprehensive model.

## Methods

### Participants and Procedure

According to Schoemann et al. (2017), to ensure the robustness of running statistics, Monte Carlo simulation should be applied to estimate the minimum sample size required for structural equation modeling. Estimation results showed that statistical efficiency was higher when sample size was larger than 250.

Following approval from the ethics committee of the Guangzhou Sport University, Guangzhou, China (protocol code: 2025LCLL-063; approval date: 22 May 2024) and three regional tournament committees, we collected athletes’ data from 48 teams participating in the 2022 China U17 Men's Basketball Competition. Afterwards, all players received the questionnaire following a standardized introductory statement about the purpose of the research. There were no right or wrong answers to the questions and all data were kept confidential. Finally, 485 (16.46 ± 0.483) valid questionnaires were collected.

This research also investigated relevant demographic variables of participants. First, all teams were affiliated with a “sports system” (n = 30) and the “education system” (n = 18). Second, the educational background of coaches was divided into two categories: a “bachelor degree or above” (n = 25) and “below the bachelor degree” (n = 23). Specifically, all coaches in the “education system” belonged to the “bachelor degree or above” category. In the “sports system” category, coaches with the “bachelor degree or above” accounted for 76.7% (n = 23) and those with “below the bachelor degree” accounted for 23.3% (n = 7). Third, the sports experience of coaches was classified as “with professional sports experience” (n = 32) or “without professional sports experience” (n = 16). In the “sports system” category, all coaches were classified as “with professional sports experience”. In the “education system” category, coaches “with professional sports experience” accounted for 11.1% (n = 2) and those “without professional sports experience” accounted for 88.9% (n = 16). The demographic characteristics of players are shown in Table 1.

**Table 1:** Description of demographic characteristics of athletes.

Variable	Category	Team Affiliation						Total	
		Sports System			Education System				
		n	%		n	%		n	%
Sports Level	Without a Sports Level Certificate	147	49.7%		112	59.3%		259	53.4%
	With a Sports Level Certificate	149	50.3%		77	40.7%		226	46.6%
Training Years	Low Training Experience (<5Y)	56	18.9%		67	35.4%		123	25.4%
	High Training Experience (≥5Y)	240	81.1%		122	64.6%		362	74.6%

Note: Y = years

### Measures

All items in the questionnaire were scored based on a 7-point system from “strongly disagree” (1 point) to “strongly agree” (7 points).

### Perceived Coach Supportive Behavior

Coach supportive behavior was measured using a method adapted from Rocchi et al. (2017), which included 12 items assessing coaches' support. In this research, a cross-cultural test, the revision of the questionnaire and confirmatory factor analysis (CFA) showed that χ^2^/df = 1.720, the root mean square error of approximation (RMSEA) = 0.039, the goodness-of-fit index (GFI) = 0.975, the incremental fit index (IFI) = 0.989, the Tucker-Lewis index (TLI) = 0.985, and the comparative fit index (CFI) = 0.989. Internal consistency of the questionnaire in our sample was α = 0.890, ranging from 0.828 to 0.850 for each subscale. The questionnaire exhibited good reliability and validity.

### Perceived Coach Controlling Behavior

Perceived coach control behavior was measured in this research using the Coach Control Behavior Questionnaire (CCBQ) developed by Bartholomew et al. (2010), which included 14 items in four dimensions. The obtained CFA test results showed that χ^2^/df = 1.421, RMSEA = 0.029, GFI = 0.971, IFI = 0.991, TLI = 0.989, and CFI = 0.991. Internal consistency of this questionnaire in the current sample was α = 0.903, ranging from 0.811 to 0.856 for each subscale. The scale had good reliability and validity.

### Satisfaction and Frustration of Basic Psychological Needs

The Chinese versions of BPNS and BPNF scales have been applied to Chinese college athletes (Li et al., 2019). The BPNS scale contained 12 items in three dimensions. In this research, CFA test results showed that χ^2^/df = 3.317, RMSEA = 0.069, GFI = 0.952, IFI = 0.971, TLI = 0.961, and CFI = 0.971. Also, the internal consistency of this questionnaire was α = 0.906 and α coefficients of the three subscales ranged from 0.818 to 0.900.

The BPNF scale consisted of three dimensions with 12 items. In this study, CFA test results revealed that χ^2^/df = 3.543, RMSEA = 0.072, GFI = 0.949, IFI = 0.963, TLI = 0.950, and CFI = 0.963. Furthermore, the internal consistency of this questionnaire was α = 0.901 and subscale Cronbach's α ranged from 0.802 to 0.870. These indicated that both BPNS and BPNF scales had good reliability and validity.

### Psychological Capital Questionnaire

The short version of the PsyCap (PCQ-12) questionnaire developed by Luthans et al. (2015), which included 12 items in four dimensions, was applied in this research to measure the PsyCap of athletes. The questionnaire was obtained from the website (https://www.mindgarden.com/136-psychological-capital-questionnaire) with research permission from the authors. CFA test results showed that χ^2^/df = 2.042, RMSEA = 0.046, GFI = 0.966, IFI = 0.984, TLI = 0.978, and CFI = 0.984. Furthermore, the internal consistency of this questionnaire was α = 0.895 and subscale Cronbach's α ranged from 0.844 to 0.883. The results proved that the scale had good reliability and validity.

### Training Engagement and Disaffection

This research revised the Rodríguez-Medellín et al.’s (2020) instrument for evaluating classroom engagement and disaffection and we applied it to the context of Chinese youth basketball training. The CFA results obtained in this research using a training engagement and disaffection scale showed that χ^2^/df = 3.680/2.341, RMSEA = 0.074/0.053, GFI = 0.952/0.971, IFI = 0.975/0.986, TLI = 0.965/0.981, and CFI = 0.974/0.986. Also, the Cronbach's α for the training engagement and disaffection scale were 0.902 and 0.898, respectively, and subscale Cronbach's α ranged from 0.876 to 0.900. These indicated that both scales had good reliability and validity.

## Results

### Common Method Deviation Test

In this research, we aggregated all scales into a one-factor model after dimensionality reduction by confirmatory factor analysis, which revealed a poor fit of the model: χ^2^/df = 5.181, RMSEA = 0.093, CFI = 0.786, and TLI = 0.762. In addition, the method of controlling for unmeasured latent factors, which added a common method bias factor to the baseline model, was employed to evaluate whether there was a significant improvement in the fit of the model (7 factors + CMV) compared to the baseline model (7 factors). The obtained results showed ΔTLI = 0.001 between the model containing the common method bias factor and the baseline model. This indicated that homogeneous variance did not significantly affect the results of this research.

### Discriminant Validity

CFA was employed to evaluate discriminant validity among the measured values of various variables in the research model. Since none of the scales used were one-dimensional, all scales were downgraded to the first-order model, the confirmatory factor model was constructed by Mplus 8.3, and the 7-factor model was considered the baseline model. The results are presented in Table 2.

**Table 2:** Discriminant validity test results for each variable (N = 485).

Model		χ^2^	df	Δχ^2^	χ^2^/df	RMSEA	CFI	TLI
baseline model	CSB,CCB,BPNS,BPNF,PsyCap,TE,TD	199.857	168	–	1.190	0.020	0.991	0.989
6-factor model a	CSB+CCB,BPNS,BPNF,PsyCap,TE,TD	433.749	174	233.892***	2.493	0.056	0.930	0.915
6-factor model b	CSB,CCB,BPNS+BPNF,PsyCap,TE,TD	380.781	174	180.924***	2.188	0.050	0.944	0.932
6-factor model c	CSB,CCB,BPNS,BPNF,PsyCap,TE+TD	268.358	174	68.501***	1.542	0.033	0.974	0.969
6-factor model d	CSB,CCB,BPNS,BPNF+PsyCap,TE,TD	402.346	174	202.489***	2.312	0.052	0.938	0.925
6-factor model e	CSB,CCB,BPNS+PsyCap,BPNF,TE,TD	386.999	174	187.142***	2.224	0.050	0.942	0.930
5-factor model	CSB+CCB,BPNS+BPNF,PsyCap,TE,TD	614.350	179	414.493***	3.432	0.071	0.882	0.861
4-factor model	CSB+CCB,BPNS+BPNF+PsyCap,TE,TD	783.036	183	583.179***	4.279	0.082	0.837	0.813
3-factor model	CSB+CCB,BPNS+BPNF+PsyCap,TE+TD	834.057	186	634.200***	4.484	0.085	0.824	0.801
2-factor model	CSB+CCB+BPNS+BPNF+PsyCap,TE+TD	958.096	188	758.239***	5.096	0.092	0.791	0.767
1-factor model	CSB+CCB+BPNS+BPNF+PsyCap+TE+TD	979.138	189	779.281***	5.181	0.093	0.786	0.762
Adding a common method bias factor to the baseline model		195.327	167	4.530*	1.170	0.019	0.992	0.990

Note: CSB = Coach Supportive Behaviors, CCB = Coach Controlling Behaviors, TE = Training Engagement, TD = Training Disaffection; * p < 0.05; ** p < 0.01; *** p < 0.001

### Descriptive Statistical and Correlation Analyses

As presented in Table 3, the descriptive statistics and correlation analysis results of various variables revealed significant correlations among particular variables (*p* < 0.01).

**Table 3:** Descriptive statistics and correlations among research variables.

	Mean	Std. Deviation	1	2	3	4	5	6	7	8	9	10	11	12
1. TA	1.390	0.488	--											
2. CEB	1.854	0.354	0.331^**^	--										
3. CSE	0.654	0.476	−0.911^**^	−0.301^**^	--									
4. ASL	0.466	0.499	−0.094^*^	−0.022	0.063	--								
5. ATY	1.746	0.436	−0.185^**^	−0.054	0.193^**^	0.421^**^	--							
6. CSB	4.195	1.323	0.335^**^	0.109^*^	−0.298^**^	0.223^**^	0.075	--	−0.557	0.548	−0.568	0.592	0.661	−0.617
7. CCB	5.643	0.748	−0.181^**^	−0.075	0.169^**^	−0.083	0.004	−0.437^**^	--	-0.518	0.601	−0.571	−0.529	0.706
8. BPNS	4.164	1.402	0.174^**^	0.041	−0.168^**^	0.102^*^	0.048	0.421^**^	−0.402^**^	--	−0.580	0.568	0.637	−0.650
9. BPNF	5.755	0.964	−0.046	−0.014	0.037	−.170^**^	−0.035	−0.442^**^	0.475^**^	−0.452^**^	--	−0.588	-0.561	0.743
10. PsyCap	5.558	0.902	0.165^**^	0.084	−0.151^**^	0.217^**^	0.122^**^	0.469^**^	−0.450^**^	0.440^**^	−0.465^**^	--	0.649	−0.715
11. TE	4.511	1.519	0.176^**^	0.039	−0.150^**^	0.134^**^	0.044	0.476^**^	−0.388^**^	0.466^**^	−0.410^**^	0.480^**^	--	−0.689
12. TD	5.094	1.234	−0.142^**^	−0.072	0.149^**^	−0.128^**^	-0.003	−0.450^**^	0.523^**^	−0.471^**^	0.555^**^	−0.529^**^	−0.477^**^	--

Note: TA = Team Affiliation, CEB = Coach Educational Background, CSE = Coach Sports Experience, ASL = Athlete Sports Level, ATY = Athlete Training Years; ** p < 0.01

### Structural Equation Modelling

**Figure 1:**
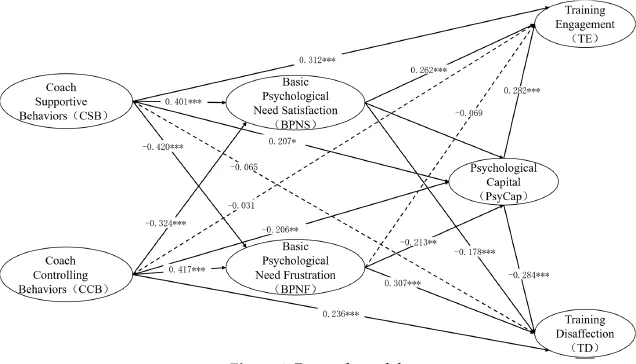
Research model.

Taking the characteristic variables of coaches and athletes as control variables, structural equation modeling results are illustrated in Figure 1. Model fitting results (χ2/df = 1.270, RMSEA = 0.024, GFI = 0.955, IFI = 0.987, TLI = 0.982, and CFI = 0.987) showed that the model had good fitness.

Supportive behavior had significant effects on engagement (β = 0.312, *p* < 0.001), although controlling behavior did not significantly affect engagement (β = −0.031, *p* = 0.707). Furthermore, controlling behavior was found to have significant effects on disaffection (β = 0.236, *p* < 0.01), but supportive behaviors did not significantly influence it (β = −0.065, *p* = 0.440) (Table 4).

**Table 4:** Path analysis results.

Path			Estimate(B)	S.E.	t	*p*	Std. Estimate (β)
CSB	→	BPNS	0.586	0.119	4.940	***	0.401
CCB	→	BPNS	−0.603	0.134	−4.515	***	−0.324
CSB	→	BPNF	−0.455	0.082	−5.532	***	−0.420
CCB	→	BPNF	0.576	0.096	6.033	***	0.417
CSB	→	PsyCap	0.209	0.087	2.397	*	0.207
CCB	→	PsyCap	−0.263	0.098	−2.674	**	−0.206
BPNS	→	PsyCap	0.142	0.048	2.971	**	0.206
BPNF	→	PsyCap	−0.197	0.072	−2.723	**	−0.213
BPNS	→	TE	0.297	0.086	3.450	***	0.262
BPNF	→	TE	−0.105	0.127	−0.825	0.410	−0.069
BPNS	→	TD	−0.162	0.062	−2.598	**	−0.178
BPNF	→	TD	0.375	0.096	3.910	***	0.307
PsyCap	→	TE	0.465	0.136	3.422	***	0.282
PsyCap	→	TD	−0.374	0.100	−3.731	***	−0.284
CSB	→	TE	0.517	0.156	3.309	***	0.312
CCB	→	TD	0.398	0.129	3.089	**	0.236
CSB	→	TD	−0.086	0.111	−0.772	0.440	−0.065
CCB	→	TE	−0.065	0.172	−0.376	0.707	−0.031

Note: * p < 0.05; ** p < 0.01; *** p < 0.001

### Mediating Effect Test

A bias corrected bootstrap was employed to explore the mediating effects of BPNs and PsyCap. In this study, 5000 Bootstrap samples were randomly adopted and 95% confidence intervals of bootstraps were calculated to estimate indirect effects.

Coach behaviors (supportive and controlling) were not able to indirectly predict training engagement through BPNF. However, the 95% confidence intervals of other mediating paths did not include 0, indicating that mediation effects were remarkable (Table 5).

**Table 5:** Results of mediation effect analysis.

Path		Std. Estimate	SE	95% CI		Ratio (%)
				Lower	Upper	
CSB→TE	Total Effect	0.553	0.104	0.349	0.757	100.0
	Direct Effect	0.312	0.132	0.051	0.579	56.4
	Total Indirect Effect	0.241	0.087	0.100	0.456	43.6
	CSB→BPNS→TE	0.105	0.051	0.030	0.242	19.0
	CSB→PsyCap→TE	0.059	0.043	0.001	0.177	10.7
	CSB→BPNF→TE	0.029	0.049	−0.060	0.140	5.2
	CSB→BPNS→PsyCap→TE	0.023	0.013	0.006	0.063	4.2
	CSB→BPNF→PsyCap→TE	0.025	0.017	0.004	0.075	4.5
CSB→TD	Total Effect	−0.373	0.093	−0.554	−0.187	100.0
	Direct Effect	−0.065	0.099	−0.252	0.137	17.4
	Total Indirect Effect	−0.308	0.090	−0.520	−0.161	82.6
	CSB→BPNS→TD	−0.071	0.040	−0.167	−0.009	19.0
	CSB→PsyCap→TD	−0.059	0.041	−0.169	−0.002	15.8
	CSB→BPNF→TD	−0.129	0.064	−0.281	−0.028	34.6
	CSB→BPNS→PsyCap→TD	−0.023	0.013	−0.063	−0.007	6.2
	CSB→BPNF→PsyCap→TD	−0.025	0.017	−0.076	−0.004	6.7
CCB→TE	Total Effect	−0.246	0.088	−0.418	−0.069	100.0
	Direct Effect	−0.031	0.098	−0.218	0.166	12.6
	Total Indirect Effect	−0.216	0.081	−0.407	−0.087	87.8
	CCB→BPNS→TE	−0.085	0.041	−0.188	−0.024	34.6
	CCB→PsyCap→TE	−0.058	0.032	−0.151	−0.014	23.6
	CCB→BPNF→TE	−0.029	0.051	−0.149	0.057	11.8
	CCB→BPNS→PsyCap→TE	−0.019	0.012	−0.057	−0.004	7.7
	CCB→BPNF→PsyCap→TE	−0.025	0.017	−0.074	−0.003	10.2
CCB→TD	Total Effect	0.524	0.094	0.333	0.705	100.0
	Direct Effect	0.236	0.113	0.022	0.464	45.0
	Total Indirect Effect	0.288	0.074	0.163	0.455	55.0
	CCB→BPNS→TD	0.058	0.032	0.009	0.139	11.1
	CCB→PsyCap→TD	0.058	0.034	0.011	0.153	11.1
	CCB→BPNF→TD	0.128	0.061	0.030	0.274	24.4
	CCB→BPNS→PsyCap→TD	0.019	0.011	0.005	0.052	3.6
	CCB→BPNF→PsyCap→TD	0.025	0.016	0.003	0.071	4.8

## Discussion

This research aimed to determine whether there was a relationship among coach behaviors, basic psychological needs, psychological capital, training engagement and disaffection in Chinese youth male basketball athletes. The results of the study confirmed a relationship between coach behaviors and athletes' TE and TD, as well as the multiple mediating mechanisms of BPNs and PsyCap in it. The majority of our hypotheses was confirmed.

### The Relationships of Coach Behavior and Engagement/Disaffection

Coach supportive behavior was found to have a significant positive effect (56.4%) on training engagement of athletes, which was consistent with previous research (Reynders et al., 2019). This was because coach autonomy support was able to stimulate self-determination motivation, which contributed to higher quality of engagement (Reynders et al., 2019). Competence support enhanced achievement-related abilities of athletes, contributed to the development of intrinsic motivation among athletes, and provided a framework for positive engagement (Fransen et al., 2018). Furthermore, when athletes perceived affection, care and respect from coaches, they developed sense of belonging, which in turn produced positive and adaptive outcomes, such as engagement (Sparks et al., 2015).

However, there was no significant correlation between coach supportive behavior and disaffection in this research, which is consistent with the findings of Van den Berghe et al. (2016). Coach supportive behaviors enable athletes to experience certain degrees of autonomy and close interpersonal relationships. Furthermore, getting clear instructions and positive feedback from the coach is more likely to be related to positive experiences of athletes rather than negative ones. Supportive and controlling behaviors are two completely opposite motivational styles and low support does not represent nor contain controlling elements (Abós et al., 2023). This could also be a reason why coach supportive behavior had no significant effect on disaffection.

On the other hand, coach controlling behavior had direct and positive effects on the training disaffection of athletes, accounting for 45.0%. Specifically, controlling coaches actively disrupt the volitional function of athletes using several strategies that are either harsh or overbearing (Aelterman et al., 2019), which make athletes more likely to display disaffected emotions and behaviors during training. Also, this research showed that coach controlling behavior did not significantly affect engagement, which further confirmed the findings of Haerens et al. (2015) and Van den Berghe et al. (2016). This might be because when athletes are faced with a controlling coach, they are absent-minded and more likely to present disaffected emotions and behaviors in training, rather than lower engagement.

### The Mediating Roles of BPNs and PsyCap

In the current study, we also found that perceived coach supportive behavior positively affected the engagement of athletes through BPNS and alleviated their disaffection through higher BPNS and lower BPNF. Psychological need satisfaction provides metal energy for engagement, paving the way for enjoyment, persistence and effort, fostering the initiative of athletes, and providing a basis for resistance to passivity, negative emotions and behaviors (Ryan and Deci, 2017).

On the other hand, controlling behaviors are indirectly linked to more disaffection and lower-quality engagement. This is mainly because the controlling environment is more likely to generate controlling motivation, resulting in high BPNF and low BPNS of athletes, making them prone to negative emotions, boredom and breeding passivity, and hindering their efforts and persistence (Sevil-Serrano et al., 2021). Therefore, cross-path test results further verified the findings of Curran et al. (2014, 2016). BPNS had unique effects on both engagement and disaffection, while BPNF only significantly influenced disaffection. This is because BPNS is not only considered to be mainly conducive to positive experience, but also can resist negative experiences by developing the psychological resources required to cope effectively, while BPNF only elicits negative responses (Deci et al., 2017; Curran and Standage, 2017).

We also found that PsyCap played a significant mediating role between coach behaviors (supportive and controlling) and engagement/disaffection. Coach supportive behavior creates positive conditions for promoting the development of athletes’ PsyCap. In turn, these positive psychological states help athletes stimulate their willingness to adopt and pursue effective behaviors, believe in their ability to cope with challenges, motivate individuals to make greater efforts, actively participate in training (Lai et al., 2020), and effectively counteract the negative experiences of stress and stressful environments (Lee et al., 2022; Vega-Díaz et al., 2025). However, athletes perceive more apathy, negative feedback and attention, punishment, threat, rejection, pressure and other negative experiences when facing controlling coaches, which in turn negatively affects the goal setting, the confidence level and self-efficacy of athletes (de Albuquerque et al., 2021), hindering the construction of psychological capital.

Finally, we found that all chain mediating paths of BPNs (satisfaction vs. frustration) and PsyCap were significant, which provides practical ways to link BPN operation mechanisms to PsyCap cultivation. Specifically, coach supportive behaviors can provide athletes with an environment that supports their autonomy, competence and relatedness by offering strategies such as giving them positive feedback, expressing interest and care for them, and providing them with the freedom to make their own choices (Alexe et al., 2023). Conversely, coaches with controlling styles demonstrate coercive, authoritarian, and pressure acts, which can prevent athletes ' BPNS, and even trigger BPNF (Su and Zhao, 2023). This proposal is coherent with the resource caravan passageways notion, which points out that people´s resources exist in an environment that either fosters/nurtures or limits/blocks resource creation or nourishment (Hobfoll et al., 2018). Thus, different coaching behaviors have an impact on psychological capital by supporting or frustrating athletes' BPNs, which in turn has an impact on athletes' attitudes, behaviors and performance (e.g., engagement, disaffection).

## Limitations and Future Directions

In this research, we discussed the effects of coach behaviors on the training engagement and disaffection of athletes and their mechanisms in the context of Chinese sports culture. Research results had important theoretical significance to understand the engagement and disaffection of Chinese youth basketball players during training, and could also provide valuable recommendations for coaches. However, limitations still exist. First, the non-experimental cross-sectional research design could not infer causal relationships among different variables. Second, only male athletes were investigated in this study due to the time constraints of the competition and the impact of the COVID-19, which restricts the generalizability of the results. Therefore, future research should explore the effects of different moderating variables (e.g., gender, age, competitive level, team vs. individual sports, etc.) on the relationship between variables considered in this study. Finally, future research should consider investigating coach training interventions and their effects on athletes' psychological outcomes to validate the causal relationship.

## Conclusions

Coach supportive behavior positively affected BPNS, PsyCap, and engagement, while coach controlling behavior induced BPNF and disaffection, which was not conducive to psychological capital. Furthermore, BPNs (satisfaction and frustration) and PsyCap exerted multiple mediating effects between coach behavior and engagement and disaffection of athletes. The research concluded that Chinese youth basketball coaches should shift their behavior style from controlling to supportive in training to stimulate the intrinsic motivation of athletes and then, increase the engagement of athletes and reduce their disaffection. At the same time, it is necessary to strengthen the cultivation of positive psychological capital among athletes to counter and buffer against adverse effects of non-adaptive environments.
